# A Comprehensive Analysis of Hungarian MODY Patients—Part I: Gene Panel Sequencing Reveals Pathogenic Mutations in *HNF1A*, *HNF1B*, *HNF4A*, *ABCC8* and *INS* Genes

**DOI:** 10.3390/life11080755

**Published:** 2021-07-27

**Authors:** Zsolt Gaál, Zsuzsanna Szűcs, Irén Kántor, Andrea Luczay, Péter Tóth-Heyn, Orsolya Benn, Enikő Felszeghy, Zsuzsanna Karádi, László Madar, István Balogh

**Affiliations:** 1 4th Department of Medicine, Jósa András Teaching Hospital, 4400 Nyíregyháza, Hungary; dr.gaal.zsolt@szszbmk.hu; 2 Division of Clinical Genetics, Department of Laboratory Medicine, Faculty of Medicine, University of Debrecen, 4032 Debrecen, Hungary; szucs.zsuzsanna@med.unideb.hu (Z.S.); madar.laszlo@med.unideb.hu (L.M.); 3 Department of Pediatrics, Jósa András Teaching Hospital, 4400 Nyíregyháza, Hungary; kantoriren@index.hu; 4 1st Department of Pediatrics, Semmelweis University, 1085 Budapest, Hungary; luczay.andrea@med.semmelweis-univ.hu (A.L.); toth-heyn.peter@med.semmelweis-univ.hu (P.T.-H.); 5 Department of Pediatrics, Szent György Hospital of Fejér County, 8000 Székesfehérvár, Hungary; bennorsolya@gmail.com (O.B.); zskaradi@mail.fmkorhaz.hu (Z.K.); 6 Department of Pediatrics, Faculty of Medicine, University of Debrecen, 4032 Debrecen, Hungary; felszeghy.eniko@med.unideb.hu

**Keywords:** MODY, monogenic diabetes, *HNF1A*, transcription factor MODY, next-generation sequencing, NGS, Hungary

## Abstract

Maturity-onset diabetes of the young (MODY) has about a dozen known causal genes to date, the most common ones being *HNF1A, HNF4A, HNF1B* and *GCK*. The phenotype of this clinically and genetically heterogeneous form of diabetes depends on the gene in which the patient has the mutation. We have tested 450 Hungarian index patients with suspected MODY diagnosis with Sanger sequencing and next-generation sequencing and found a roughly 30% positivity rate. More than 70% of disease-causing mutations were found in the *GCK* gene, about 20% in the *HNF1A* gene and less than 10% in other MODY-causing genes. We found 8 pathogenic and 9 likely pathogenic mutations in the *HNF1A* gene in a total of 48 patients and family members. In the case of *HNF1A*-MODY, the recommended first-line treatment is low dose sulfonylurea but according to our data, the majority of our patients had been on unnecessary insulin therapy at the time of requesting their genetic testing. Our data highlights the importance of genetic testing in the diagnosis of MODY and the establishment of the MODY subtype in order to choose the most appropriate treatment.

## 1. Introduction

In addition to the more common type 1 and 2 diabetes mellitus (DM), monogenic forms also exist. The major groups of these monogenic forms can be classified into two main categories: the transient or permanent neonatal form (NDM) presents before the age of six months whereas maturity-onset diabetes of the young (MODY) generally has an age of onset before 25 years, but there are exceptions [1,2]. Furthermore, diabetes can co-occur with several other symptoms as a form of rare diabetes-associated syndromes [3]. More than 20 causal genes have been linked to monogenic diabetes so far, the strongest evidence and highest prevalence having the transcription factors *HNF1A*, *HNF4A*, *HNF1B* and the *GCK* gene encoding the glucokinase enzyme [4].

In this article, we focus on MODY (OMIM # 606391), an autosomal dominant form of diabetes caused by heterozygous mutations in one of the MODY-associated genes resulting in beta-cell dysfunction [5]. First described in the 1970s [6,7], this form of diabetes is clinically and genetically heterogeneous [8]. The phenotypic presentation depends on which gene harbours the disease-causing mutation, the most characteristic one being hyperglycaemia as a result of the disruption in insulin production/release processes [9]. Certain types of gene mutations can cause long-term complications, especially if left untreated, whereas others result in patients requiring only oral medication instead of insulin or no treatment at all [9].

The five major MODY criteria are: (1) age of onset before 25 years; (2) autosomal dominant pattern of inheritance; (3) not requiring insulin therapy or detectable C-peptides; (4) beta-cell dysfunction with normal insulin levels; and (5) the absence of the obesity which is generally associated with diabetes [10].

Transcription factor MODY (MODY1, MODY3 and MODY5): Whereas *GCK*-MODY (presented in Part II of this article) presents as a mild form of diabetes, patients with transcription factor MODY show more severe phenotypes. The most common transcription factors that cause MODY when mutated belong to the hepatocyte nuclear factor (HNF) family (HNF4A, HNF1A and HNF1B), resulting in MODY1, MODY3 and MODY5, respectively, but other causal transcription factor genes have also been described [11].

The *HNF* genes play an important role in liver development and function but in MODY it is their pancreatic activity that is primarily affected [11,12]. *HNF1A* and *HNF4A* mutations both cause progressive beta-cell dysfunction as they play a vital role in beta-cell development. This results in a similar clinical picture and increases the risk of later-onset complications [11]. Compared to *GCK*-MODY, in addition to hyperglycaemia, *HNF1A*- and *HNF4A*-MODY present with more characteristic phenotypes, such as polyuria, polydipsia and weight loss [13]. Both types might present with neonatal transient hyperinsulinaemic hypoglycaemia followed by a progressive insulin secretory defect later in life [14].

*HNF1A*-MODY is mainly the result of haploinsufficiency due to loss-of-function mutations [15]. The *HNF1A* gene has three isoforms generated from the same promoter by alternative splicing and it has three functional domains: an N-terminal dimerization domain, a DNA-binding domain and a C-terminal transactivation domain [16]. This gene is considered to be polymorphic; mutations have been described in every domain of the gene, with no mutational hot-spots [17]. To date, about 500 disease-causing small scale *HNF1A* mutations have been reported in the professional version of the HGMD (Human Gene Mutation Database, version 2021_1) associated with the MODY phenotype, the majority of them being missense alterations resulting in amino acid change. More than 80% of *HNF1A* mutations have been reported in exons 1–6 of 10, affecting all three isoforms of the gene and having a lower average age of diagnosis as compared to those who have mutations in exons 8–10. Patients with truncating *HNF1A* mutations generally have a younger age of onset compared to those who have a missense mutation, suggesting phenotype variability of MODY3 patients based on mutation type and location [9,18].

Mutations in *HNF1A* alter the expression of glucose transporter proteins and enzymes involved in glucose metabolism and also result in a decrease in the amount of insulin produced [9,19]. The presenting hyperglycaemia might be deteriorating and progressive, with a risk of developing complications in the long term similar to type 1 and 2 diabetes. Rigorous glucose control is required to prevent the development of long term complications [19,20]. HNF1A is involved in the glucose reuptake in the kidneys as well; *HNF1A* mutations thus result in renal glycosuria because of the low renal threshold for glucose. This usually precedes the beta-cell insulin secretion defect for years and it is not characteristic of *HNF4A*-MODY [17]. *HNF1A*-MODY has age-dependent high penetrance; almost 63% of the patients with *HNF1A* mutations develop symptoms by the age of 25, 93.6% by the age of 50 years, and 98.7% by the age of 75 years [21,22].

HNF4A is involved in the regulation of glucose transport and metabolism genes [23] and also regulates the expression of several proteins involved in lipid metabolism [24]. *HNF4A*-MODY is associated with macrosomia at birth [14].

Low dose sulphonylurea is the best first-line treatment in both MODY1 and MODY3 cases as it regulates blood glucose level, decreases glycosylated haemoglobin and prevents future insulin dependencies [25]. However, with the progression of diabetes, some patients may require additional insulin therapy as well [19].

MODY5 is caused by mutations in the hepatocyte nuclear factor-1 beta (*HNF1B*) gene encoding a transcription factor involved in the early development of the pancreas, kidney, liver, lungs, gut and genitourinary tract. As a result, *HNF1B*-MODY patients can develop abnormalities in all of these organs in addition to hyperglycaemia [19], the symptoms of which often precede the diabetes. They can be characterized by pancreas hypoplasia resulting in beta-cell dysfunction and reduced insulin secretion [21]. About 50% of the patients present with hypomagnesaemia and hypokalaemia as well [26,27]. By the age of 45, patients usually develop renal dysfunction and about half of them progress to end-stage renal failure [19]. In contrast with *HNF4A*-MODY, *HNF1B* mutations can reduce birthweight by up to 900 g [19,28].

*HNF1B* loss-of-function mutations result in haploinsufficiency, which is responsible for the development of the disease. Large differences can be seen in phenotypic presentation and age of onset even in the same family. Furthermore, half of the patients with *HNF1B*-MODY have de novo mutations, and half of them also carry a large heterozygous deletion, which is in contrast with other types of MODY, where the mutations are mostly inherited from one of the parents and are usually a small-scale missense mutation [21].

In contrast with other MODY types, *HNF1B*-MODY patients do not respond to sulphonylurea, they usually require early insulin therapy and nephropathy management may also be necessary [19].

In these two accompanying papers, we describe the results of a 10-year genetic and clinical analysis of Hungarian MODY patients. Being the sole genetic centre in the country, our results represent the data of the entire Hungarian cohort of MODY.

## 2. Materials and Methods

### 2.1. Patients

A total of 450 unrelated index patients with suspected MODY diagnosis and their 202 family members have been referred to our laboratory for genetic testing from all around Hungary. All participants or their guardians gave informed consent to genetic testing according to national regulations.

### 2.2. Methods

Genomic DNA was isolated from peripheral blood leukocytes using the QIAamp Blood Mini kit (Qiagen GmbH, Hilden, Germany).

In the case of 102 index patients, Sanger sequencing of the *GCK*, *HNF1A* or *HNF4A* genes was performed using the BigDye Terminator v3.1 Cycle Sequencing kit (Applied Biosystems, Foster City, CA, USA) according to the manufacturer’s protocol.

Bidirectional pyrosequencing with a minimum coverage of 40× was performed on Roche GS Junior 454 pyrosequencing system (Roche 454 Life Sciences, Branford, CT, USA) in the case of 33 index patients.

We sequenced 311 index patient samples on Illumina Miseq or NextSeq 550 (Illumina, San Diego, CA, USA) sequencer systems in 2 × 150 cycle (or 2 × 250 cycle in case of the MODY MASTR kit) paired-end mode. We used 3 different library preparation methods before sequencing. The MODY MASTR kit (Multiplicom, Niel, Belgium) was used to examine 7 genes in the case of 76 index patients. A custom-made and enrichment-based DNA library preparation kit (Qiagen, GmbH, Hilden, Germany) containing 17 genes was used in the case of 164 index patients, and another custom-designed gene panel (Twist Bioscience, South San Francisco, CA, USA) was used, examining 18 genes in case of 69 and 20 genes in case of 6 index patients (Supplementary Table S1). In the case of Illumina sequenced data, data analysis was performed using the NextGene software (SoftGenetics, State College, PA, USA).

MLPA (Multiplex ligation-dependent probe amplification) was performed in the case of 32 index patients (as a single test in the case of 4 index patients and in addition to one of the above-mentioned methods in the case of 28 index patients) using SALSA MLPA Probemix P241 MODY Mix 1 and/or SALSA MLPA Probemix P357 MODY Mix 2 (MRC Holland, Amsterdam, The Netherlands) according to the manufacturer’s protocol.

The testing method(s) used in the case of every index patient is described in Supplementary Table S2.

Cascade testing was performed in 202 family members usually by targeted Sanger sequencing of the respective exon of the MODY-causing gene in which their relative had a possibly pathogenic mutation.

### 2.3. Variant Confirmation

All variants obtained with next-generation sequencing that were suspected to be disease-causing were validated by Sanger sequencing. Furthermore, when the amplicon’s minimum coverage was <40× in the NGS data, the respective exons were also sequenced using the Sanger method.

### 2.4. Variant Filtering and Interpretation

All detected variants having a MAF > 0.01 (minor allele frequency) in the gnomAD population database were filtered. The remaining variants were classified according to the ACMG standards and guidelines [29,30]. A web-based interpretation tool, Franklin (Genoox) [31] was used to assist the classification. HGMD Professional and ClinVar databases were also used in variant interpretation.

### 2.5. Clinical Data Collection

Clinical data of patients and family members having a ‘pathogenic’ (‘P’) or ‘likely pathogenic’ (‘LP’) mutation in one of the MODY-causing genes were collected from their application form, sent and filled out by their clinician at the time of requesting the genetic testing. The MODY Probability Calculator (https://www.diabetesgenes.org/ (accessed on 20 March 2021)) was used to calculate the probability of the patient having MODY when all information required was available and the patient was under the age of 35, as the calculator cannot be used in case of patients older than that.

## 3. Results

From the 450 index patients examined, 132 tested positive for a variant classified as ‘P’ or ‘LP’ in one of the MODY-causing genes with a total of 89 mutations, meaning a roughly 30% of positivity rate. More than 70% (65/89) of the mutations were found in the *GCK* gene (described in Part II of this article), around 20% of the mutations (17/89) were found in the *HNF1A* gene and the remaining roughly 10% (7/89) in other MODY-causing genes (Table 1). Every mutation detected was in a heterozygous form.

Furthermore, 202 family members of the index patients harbouring a MODY-causing ‘P’ or ‘LP’ mutation were also examined, half of which (95/202) tested positive for a MODY-causing mutation. This means that a total of 227 patients were diagnosed with MODY in the examined cohort and given a clinically relevant molecular genetic diagnosis as well. Three-quarters (72/95) of the positively tested family members had a mutation in the *GCK* gene (presented in Part II of this article), around 20% (18/95) in the *HNF1A* gene and 5% (5/95) in other MODY-causing genes.

### 3.1. HNF1A Families

**Figure 1:**
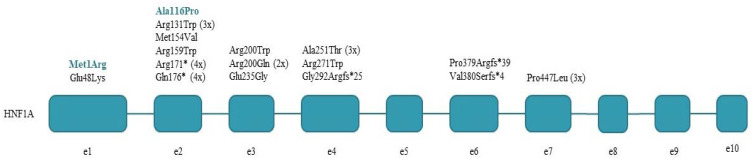
*HNF1A* mutations detected in the index patients. Novel mutations are shown in colour.

We have identified 17 different pathogenic (9) and likely pathogenic (8) *HNF1A* mutations in 30 index patients and their 18 family members, summarized in Table 2 and Figure 1. Six mutations were found in more than one apparently unrelated families, the most frequent ones being two nonsense mutations, p.Arg171* and p.Gln176*, found in 4-4 families, respectively. Two of 17 mutations (12%) are novel ones; the rest have been described in the literature previously. We found that 70% of the detected *HNF1A* mutations are missense mutations, with Arg-Trp change being the most frequent one (4 of 12). All but one mutation was detected in exons 1–6 of the *HNF1A* gene, this being in accordance with the more than 80% reported in the literature in the common part of the gene in all three isoforms. A total of 5 truncating mutations were found among the 17 mutations: 2 nonsense and 3 frameshift ones.

Clinical data of index patients and their family members that harbour an *HNF1A* mutation are presented in Supplementary Table S3. Obesity is not characteristic of these patients; few of them have developed any complications so far. HbA1c level data were available in 29 of 48 cases, but they should be handled with caution as generally the patients were already under treatment at the time of requesting their genetic testing. Six of the 29 patients have an HbA1c level lower than 6.1% and 23 have an elevated HbA1c level. The patients were diagnosed with diabetes at different ages, and the majority of the patients have received a proper molecular genetic diagnosis only several years later.

The recommended first-line treatment in the case of *HNF1A*-MODY is low dose sulphonylurea. We had information regarding treatment in the case of 35 *HNF1A*-MODY patients, but only 5 of them had received sulphonylurea treatment (one of which was combined with metformin) at the moment of requesting their genetic testing and 21 of them had already been on insulin therapy (Table 3), generally as of their diagnosis. This suggests that many patients do not receive proper treatment in the absence of a proper molecular genetic diagnosis. We do not have any information regarding therapy following the genetic diagnosis but according to the literature, insulin therapy can be switched to sulphonylurea treatment in these cases.

With family member screening, we detected additional diabetic family members in 12 families. Nine members were still presymptomatic.

### 3.2. Mutations in Other MODY Genes

Table 4 shows the mutations we detected in MODY-causing genes other than *HNF1A* or *GCK*. Mutations detected in the *HNF1B* gene are large scale deletions of four exons or the whole gene, this being in accordance with the literature data that copy number variations are characteristic of the *HNF1B* gene.

Supplementary Table S4 presents the clinical data of these index patients and their family members.

## 4. Discussion

In our cohort examining patients from all over Hungary, 227 patients were diagnosed with MODY in about 10 years, having a 30% positivity rate, with a 20% mutation rate in the *HNF1A* gene and 70% in the *GCK* gene (see Table 1 in Part II of this article). Our result is roughly similar to the prevalence of *GCK*-MODY and *HNF1A*-MODY described in other populations in Europe. For example, a screening study in Poland reports a positivity rate of 40%, in which in 96% of the cases the *GCK* gene was responsible for the MODY phenotype [47]. An Italian study of children with incidental hyperglycaemia reports a 70% positivity rate, *GCK*-MODY being the most common with 90% [48]. In Greece, the GCK-MODY ratio is reported to be somewhat lower with only 54% and *HNF1A*-MODY with 12% [49]. A very recent Greek study puts the diagnostic rate at 20% [50]. These data show that the inclusion criteria for testing, the methodology used (i.e., number of analysed genes if gene panel testing is applied and whether copy number variation analysis is performed or not) might significantly affect the pickup rate.

It is worth mentioning that we cannot exclude the MODY diagnosis of patients in which we could not detect any pathogenic or likely pathogenic mutations, as there are other MODY-causing genes which we have not tested. Moreover, these patients could have a disease-causing mutation in the intronic, regulatory or promoter region of one of the genes examined that we have not sequenced. It is also possible that they harbour a mutation that we know very little about, and it is classified as a variant of uncertain significance.

The variants found in the *ABCC8* and *KCNJ11* genes have been published in cases with neonatal diabetes phenotype [42,46]. However, other, very similar mutations in the *KCNJ11* gene, such as the p.Glu227Lys—which is located only two amino acid residues upstream to our mutation and the same missense alteration—have been previously described in both neonatal diabetes and MODY cases [51]. The *KCNJ11* c.685G > A, p.Glu229Lys (Family 315) is indeed very interesting as the father of the index patient carries the mutation, too, and his diabetes was diagnosed at the age of 40 years while the diabetes of the index patient was diagnosed at the age of 13, suggesting a variable expressivity of the alteration, probably affected by other genetic and/or environmental factors.

Generally, these MODY positive patients had already been diagnosed with diabetes years before their genetic testing was requested, and MODY was suspected only years later, which resulted in the incorrect treatment of several patients. In many cases, MODY patients are misclassified as having Type 1 diabetes before receiving a MODY diagnosis. All of them were on insulin treatment. According to our knowledge, most of them can be transferred to successful sulphonylurea treatment.

We would like to emphasize the importance of the molecular genetic diagnosis, which can result in a change in the therapy. Although patients with *HNF1A* mutations usually need sulphonylurea treatment, having to take an oral antidiabetic drug makes their life much easier than the daily insulin injections, which cause them unnecessary discomfort in their everyday life, and improves the quality of life substantially and decreases their expenditure on treatment.

We also diagnosed family members with *HNF1A*-MODY before they started to show any symptoms of diabetes, some of them still being in early childhood. Their blood glucose levels should be closely monitored, and their therapy started when necessary, to prevent the development of long-term complications. We would also like to emphasize the importance of cascade testing as to identify all MODY patients in the families.

## Figures and Tables

**Table 1:** Distribution of the Pathogenic/Likely pathogenic mutations found in MODY-causing genes.

Gene	Number of ‘P’/‘LP’ Mutations Found
*GCK*	65 (73.0%)
*HNF1A*	17 (19.1%)
Other MODY-causing gene ^1^	7 (7.9%)

^1^ *ABCC8*, *HNF1B*, *HNF4A*, *INS*, *KCNJ11;* P: Pathogenic; LP: Likely Pathogenic.

**Table 2:** Pathogenic (8) and Likely Pathogenic (9) mutations found in the *HNF1A* gene.

Nucleotide Change	Protein Change	Exon/Intron	Function	ACMG	ACMG Evidence	ClinVar	GnomAD Alleles (MAF)	Pr/FM	Family ID	Novel/Known	Reference
**c.2T > G**	**p.Met1Arg**	**exon 1**	**Missense**	**Pathogenic**	**PM2 (2); PP1 (3); PS1 (3); PVS1 (2)**	**N/A**	**N/A**	**1/1**	**F259**	**Novel**	
c.142G > A	p.Glu48Lys	exon 1	Missense	Likely pathogenic	PM1 (2); PM2 (2); PP2 (1); PP3 (1)	Uncertain significance (2)	22 (0.00009052)	1/0	F234	Known	[32]
**c.346G > C**	**p.Ala116Pro**	**exon 2**	**Missense**	**Likely** **pathogenic**	**PM1 (2); PM2 (2); PP2 (1); PP3 (1)**	**N/A**	**N/A**	**1/0**	**F155**	**Novel**	
c.391C > T	p.Arg131Trp	exon 2	Missense	Pathogenic	PM1 (2); PM2 (2); PM5 (1); PP2 (1); PP3 (1); PP5 (3)	Pathogenic (3)	N/A	3/4	F007, F012, F228	Known	[33]
c.460A > G	p.Met154Val	exon 2	Missense	Likely pathogenic	PM1 (2); PM2 (2); PP2 (1); PP3 (1)	N/A	N/A	1/0	F128	Known	[16]
c.475C > T	p.Arg159Trp	exon 2	Missense	Pathogenic	PM1 (2); PM2 (2); PM5 (2); PP2 (1); PP3 (1); PP5 (3)	Pathogenic (2)	1 (0.000003978)	1/0	F204	Known	[34]
c.511C > T	p.Arg171*	exon 2	Nonsense	Pathogenic	PM2 (2); PP1 (2); PP5 (3); PVS1 (4)	Pathogenic (2)	0	4/3	F095, F283, F361, F380	Known	[35]
c.526C > T	p.Gln176*	exon 2	Nonsense/ Splice	Pathogenic	PM2 (2); PP1 (2); PP5 (3); PVS1 (4)	Pathogenic (2)	1 (0.000003994)	4/1	F098, F297, F302, F394	Known	[36]
c.598C > T	p.Arg200Trp	exon 3	Missense	Likely pathogenic	PM1 (2); PM2 (2); PM5 (2); PP2 (1); PP3 (1); PP5 (1)	Likely pathogenic (1)	N/A	1/1	F423	Known	[34]
c.599G > A	p.Arg200Gln	exon 3	Missense	Pathogenic	PM1 (2); PM2 (2); PM5 (1); PP2 (1); PP3 (1); PP5 (3)	Pathogenic/ Likely pathogenic (3)	1 (0.000006572)	2/0	F159, F287	Known	[37]
c.704A > G	p.Glu235Gly	exon 3	Missense	Likely pathogenic	PM1 (2); PM2 (2); PP2 (1); PP3 (1); PP5 (2)	Likely pathogenic (1)	N/A	1/1	F474	Known	[38]
c.751G > A	p.Ala251Thr	exon 4	Missense	Pathogenic	PM1 (2); PM2 (2); PP1 (3); PP2 (1); PP3 (1)	N/A	N/A	3/4	F020, F333, F508	Known	[39]
c.811C > T	p.Arg271Trp	exon 4	Missense	Pathogenic	PM1 (2); PM2 (2); PM5 (2); PP2 (1); PP3 (1); PP5 (3)	Pathogenic (2)	0	1/0	F452	Known	[34]
c.872dupC	p.Gly292Argfs*25	exon 4	Frameshift	Pathogenic	PP1 (2); PP5 (3); PVS1 (4)	Pathogenic (8)	N/A	1/1	F025	Known	[40]
c.1136_1137delCT	p.Pro379Argfs*39	exon 6	Frameshift	Pathogenic	PM2 (2); PP1 (2); PP5 (3); PVS1 (4)	Pathogenic (1)	N/A	1/2	F141	Known	[40]
c.1137delT	p.Val380Serfs*4	exon 6	Frameshift	Pathogenic	PM2 (2); PP5 (3); PVS1 (4)	Pathogenic (4)	N/A	1/0	F104	Known	[41]
c.1340C > T	p.Pro447Leu	exon 7	Missense	Likely pathogenic	PM2 (2); PP2 (1); PP3 (1); PP5 (3)	Pathogenic (3)	3 (0.00001204)	3/0	F096, F366, F430	Known	[40]

*HNF1A* reference sequence: NM_000545.5, novel mutations are shown in bold. ACMG: shows the classification of the mutation based on the ACMG guidelines; ACMG evidence: the criteria and their strength used for the ACMG classification, as follows: (1) supporting, (2) moderate, (3) strong, (4) very strong, (5) stand-alone; ClinVar: the classification of the mutation according to ClinVar, with the number of submissions in brackets; gnomAD MAF: minor allele frequency of the mutation in the gnomAD database; Pr/FM: number of probands /their family members the mutation was found in; Family ID: identification of the families the mutation was found in.

**Table 3:** Treatment of HNF1A-MODY patients prior to the genetic diagnosis.

Therapy	Number of Patients
Insulin	20
OAD-sulphonylurea	4
OAD-metformin	3
OAD	1
Combined ^1^	2
Diet	4
No treatment	1
N/A	13

^1^ OAD–metformin and sulphonylurea; OAD metformin and insulin. OAD: oral antidiabetic drug.

**Table 4:** Pathogenic and likely pathogenic mutations found in other MODY-causing genes.

Gene	Nucleotide Change	Protein Change	Exon/ Intron	Function	ACMG	ACMG Evidence	ClinVar	GnomAD Alleles (MAF)	Pr/FM	Family ID	Novel/Known	Reference
*ABCC8*	c.643G > A	p. Val215Ile	exon 5	Missense	Likely pathogenic	PM1 (1); PM2 (2); PP1 (1); PP2 (1); PP3 (1)	N/A	N/A	1/2	F196	Known	[42]
***ABCC8***	**c.3988 + 1G > A**	**Splice**	**intron 32**	**Splicing**	**Likely pathogenic**	**PM2 (2); PVS1 (4)**	**N/A**	**N/A**	**1/0**	**F305**	**Novel**	
*HNF1B*	del e1–e4			Copy Number Variation	Pathogenic				2/1	F091, F172	Known	[43]
*HNF1B*	del e1–e9			Copy Number Variation	Pathogenic				3/0	F076, F372, F388	Known	[44]
*HNF4A*	c.869G > A	p. Arg290His	exon 8	Missense	Likely pathogenic	PM2 (2); PP3 (1); PP5 (1)	Uncertain significance (2)	1 (0.000004031)	2/1	F097, F192	Known	[45]
***INS***	**c.128G > A**	**p. Cys43Tyr**	**exon 1**	**Missense**	**Likely pathogenic**	**PM1 (1); PM2 (2); PM5 (1); PP2 (1); PP3 (1)**	**N/A**	**N/A**	**1/0**	**F284**	**Novel**	
*KCNJ11*	c.685G > A	p. Glu229Lys	exon 1	Missense	Likely pathogenic	PM1 (1); PM2 (2); PP2 (1); PP3 (1); PP5 (1)	Pathogenic (1)	N/A	1/1	F315	Known	[46]

Reference sequences: *ABCC8* NM_000352.3, *HNF4A* NM_000457.4, *INS* NM_000207.2, *KCNJ11* NM_000525.3, novel mutations are shown in bold. ACMG: shows the classification of the mutation based on the ACMG guidelines; ACMG evidence: the criteria and their strength used for the ACMG classification, as follows: (1) supporting, (2) moderate, (3) strong, (4) very strong, (5) stand-alone; ClinVar: the classification of the mutation according to ClinVar, with the number of submissions in brackets; gnomAD MAF: minor allele frequency of the mutation in the gnomAD database; Pr/FM: number of probands/their family members in whom the mutation was found; Family ID: identification of the families in which the mutation was found.

## Data Availability

The data presented in this study are available on request from the corresponding author.

## Supplementary Materials

The following are available online at https://www.mdpi.com/article/10.3390/life11080755/s1, Table S1. The list of genes examined with the different library preparation kits; Table S2. Methods used for testing the index patients; Table S3. Clinical data of patients with HNF1A mutation; Table S4. Clinical data of patients having a mutation in other MODY-causing genes.
